# Reference intervals for hematology and biochemistry in juvenile Eastern spot-billed ducks (*Anas zonorhyncha*) and validation of an automated hematology analyzer for avian blood analysis

**DOI:** 10.1371/journal.pone.0334942

**Published:** 2025-10-31

**Authors:** Ockju Im, Sung-Ryong Kim, Ki-Jeong Na, Kyung-Duk Min, Dong-Hyuk Jeong

**Affiliations:** 1 Department of Wildlife and Conservation Medicine, College of Veterinary Medicine, Chungbuk National University, Cheongju, Republic of Korea; 2 Wildlife Center of Chungbuk, Cheongju, Republic of Korea; 3 Department of Veterinary Laboratory Medicine, College of Veterinary Medicine, Chungbuk National University, Cheongju, Republic of Korea; 4 Department of Veterinary Epidemiology, College of Veterinary Medicine, Chungbuk National University, Cheongju, Republic of Korea; Benha University, EGYPT

## Abstract

The Eastern spot-billed duck (*Anas zonorhyncha*) is a common migratory waterfowl widely distributed throughout East Asia. However, the lack of species-specific reference intervals, defined as the normal range of values derived from a healthy population for clinical interpretation, hampers accurate health assessment and clinical diagnostics in this species. This is further complicated by hematologic characteristics of avian blood, such as nucleated red blood cells and thrombocytes, which pose challenges to conventional automated measurement systems optimized for mammals. This study aimed to establish hematological and biochemical reference intervals for juvenile Eastern spot-billed ducks and to evaluate the accuracy of an automated hematology analyzer (XN-1000V, Sysmex, Japan), which newly supports avian blood analysis, compared to traditional manual counting methods. Blood samples were collected from 55 clinically healthy juvenile ducks. The established reference intervals were largely consistent with those reported in related *Anas* species, though Eastern spot-billed ducks exhibited a heterophil-predominant leukogram. No significant differences were observed between sexes. Comparison between manual and automated methods revealed statistically significant differences in most hematological parameters; however, most of these differences were not clinically relevant, except for thrombocyte counts. Moderate correlations were found for key parameters, including WBC, RBC, hemoglobin, PCV, and MCV, suggesting potential clinical applicability of the automated analyzer with some limitations. This study provides the first species-specific hematologic and biochemical reference intervals for juvenile Eastern spot-billed ducks under semi-captive conditions, offering valuable baseline data for clinical assessments in this subset of the population. Additionally, it highlights the need for further optimization of XN-1000V analyzers for reliable and precise application in avian diagnostics.

## Introduction

The Eastern spot-billed duck (*Anas zonorhyncha*) is a dabbling duck species commonly found across East Asia, including Russia, Mongolia, China, Taiwan, Japan and Korea [1]. In South Korea, Eastern spot-billed ducks can be easily observed in diverse habitats such as rivers, rice paddies, streams, and shores [2]. While some individuals migrate from northeastern China and eastern Russia in the autumn after breeding, many are year-round resident population, with breeding within South Korea observed since the 1960s [1]. Genetically, the Eastern spot-billed duck is closely related to the mallard (*Anas platyrhynchos*), the wild ancestor of domestic ducks (*Anas platyrhynchos domesticus*), and frequent hybridization between the two species has been reported in regions where their distributions overlap [3,4]. One notable characteristic of the Eastern spot-billed duck is its large population size, and the species is currently classified as “Least Concern” (LC) on the IUCN Red List [5]. Owing to its abundance, it is also one of the most frequently rescued bird species in South Korea, with 700–900 individuals rescued each year across 16 wildlife rehabilitation centers nationwide [6].

Despite its abundance and ecological significance, previous research on the Eastern spot-billed duck has primarily focused on phylogenetic classification, ecological traits, and surveillance of infectious diseases [2–4,7,8]. For example, several studies have employed GPS-based migration tracking and pathogen screening to examine the species’ role in the spread of avian diseases, including highly pathogenic avian influenza (HPAI) [2,7,8]. However, little attention has been paid to the species’ baseline physiological parameters, such as hematology and biochemistry reference intervals. This lack of data limits the ability to conduct accurate health assessments and disease diagnosis in this species.

Reference intervals are important tools for interpreting hematologic and biochemical parameters, supporting disease diagnosis and monitoring in both clinical and research settings [9]. These parameters have been studied in mallards as well as in various other *Anas* species, including various breeds of domestic ducks [10–20]. However, many of these studies do not fully comply with the ASVCP (American Society for Veterinary Clinical Pathology) guidelines for establishing reference intervals, due to limitations such as insufficient sample sizes, lack of documented health status of the animals studied, or the use of inappropriate statistical methods [21]. This highlights the ongoing need to establish robust reference intervals in duck species. Furthermore, previous studies across various avian species have shown that while hematologic characteristics may exhibit phylogenetic relatedness, they also differ between species, and even among closely related taxa depending on the latitude of their habitat [22–24]. This underscores the necessity of establishing species-specific reference intervals for the Eastern spot-billed duck to improve diagnostic precision.

Moreover, avian hematology presents challenges due to the presence of nucleated red blood cells and thrombocytes, which complicates performing complete blood cell counts (CBC) using impedance- or optical-based automated hematology analyzers designed for mammals. As a result, manual counting methods still remain the primary approach for avian blood analysis [25,26]. However, this method is both labor-intensive and time-consuming, and subject to considerable inter-observer variability, potentially limiting its reproducibility and efficiency in both clinical and research contexts [26]. Various approaches have been explored to automate avian hematology, and among them, image-based analysis of blood smear slides enhanced by deep learning algorithms has emerged as a representative method [27–29]. While this technique offers a major advantage in terms of cost-effectiveness, it is limited by its dependence on smear quality, difficulty in quantifying absolute cell counts, and reduced generalizability due to interspecies variation in blood cell morphology. Several studies have also investigated the use of automated hematology analyzers equipped with flow cytometry, such as the Cell-Dyn 3500 and 3700 (Abbott Laboratories, USA), for performing CBC and differential leukocyte counts in avian blood [30–32]. Studies reported that total leukocyte counts and other CBC parameters yielded reliable results [32]. However, these devices often demonstrate limited accuracy in differentials of white blood cells, making them less suitable for detailed leukocyte analysis in avian blood [30,31]. Furthermore, avian blood analysis is not officially supported by these analyzers, requiring some customization prior to use [32].

The XN-1000V analyzer (Sysmex, Japan) is an automated hematology analyzer that officially supports avian blood analysis via the PLT-F channel, which is based on fluorescence flow cytometry [33,34]. The PLT-F channel enables the identification and differentiation of nucleated blood cell populations, including red blood cells, white blood cells, and platelets, in non-mammalian species using dedicated fluorescent dyes [34]. Recent studies have applied the analyzer to non-mammalian species such as Hermann’s tortoise (*Testudo hermanni*) and rainbow trout (*Oncorhynchus mykiss*), demonstrating good agreement with manual methods and confirming its utility in those taxa [34,35]. However, to the best of the authors’ knowledge, its performance in avian hematology has not yet been validated. This study aims to address these gaps by (1) establishing species-specific hematologic and biochemical reference intervals for juvenile Eastern spot-billed ducks and (2) evaluating the validity of the XN-V automated hematology analyzer by comparing its results with those obtained using traditional manual methods. Through this approach, we aim to contribute new evidence for the applicability of automated hematology in avian species while providing rigorously derived reference values that adhere to ASVCP guidelines.

## Materials and methods

### 1. Study subjects and blood sampling

This study was conducted on healthy juvenile Eastern spot-billed ducks (*Anas zonorhyncha*) that were housed at the Chungbuk Wildlife Center and Busan Wildlife Treatment Center in South Korea between August 2023 to September 2024. All individuals had been rescued within seven days of hatching due to parental loss or entrapment in artificial structures, and showed no signs of trauma or illness at the time of rescue. The ducks were reared in semi-natural outdoor enclosures designed to resemble their natural habitat. During captivity, they were provided with a daily diet consisting of chick feed, napa cabbage, water celery, live mealworms, superworms, frozen mosquito larvae, and calcium supplements, with *ad libitum* access to water. Ducks were included in the study once they reached 3–4 months of age, at which point they were fully feathered, and capable of flight. Prior to blood collection, medical records were reviewed to confirm the absence of disease history, and physical examinations were performed. During the examinations, body weight was measured and body condition score (BCS) were assessed. The BCS was determined by palpating the pectoral muscles adjacent to the keel bone and was rated on a five-point scale ranging from 1 to 5 [36]. Scores from two independent assessors were averaged to enhance objectivity. The individuals included in the study weighed between 0.7 and 1.1 kg and had BCS values ranging from 2 to 4. Capture was performed in outdoor enclosures using hand-held nets. Ducks were then brought indoors and allowed to stabilize prior to sampling. During physical examination and blood collection, each duck was manually restrained using a towel, without chemical sedation. Individuals showing signs of excessive agitation or hyperventilation were excluded from sampling due to ethical considerations and the potential influence of stress on hematologic parameters. A total of 55 ducks met the inclusion criteria and were ultimately included in the study. All procedures involving animals were approved by the Institutional Animal Care and Use Committee (IACUC) of Chungbuk National University (Approval Nos. CBNUA-2164-23-01 and CBNUA-24-0050-01) and were conducted in accordance with ethical guidelines for animal research.

Blood samples were collected from the medial metatarsal vein using a 3 mL syringe equipped with a 24-gauge needle between 10:00 AM and 12:00 PM to minimize diurnal effects. A total of 2 mL of blood was drawn from each individual and divided into two tubes: 0.5 mL into an K_2_ ethylenediaminetetraacetic acid (EDTA) microtainer tube (BD Microtainer, Becton, Dickinson and Company, USA) and 1.5 mL into a serum separation tube (BD Vacutainer^®^, Becton, Dickinson and Company, USA). The samples were mixed thoroughly and immediately stored at 4°C. Serum was obtained by centrifuging the blood in serum separation tube at 3,000 g for 15 minutes and subsequently transferred to microcentrifuge tubes for storage. All hematological and serum biochemical analyses were conducted within 12 hours of blood collection. Automated hematology analyses were performed within 6 hours post-collection.

### 2. Hematological and biochemical analysis

**1) Complete blood cell count (CBC) using manual methods:** A total of thirteen parameters were analyzed using the manual method, including total white blood cell count (WBC), total red blood cell count (RBC), packed cell volume (PCV), hemoglobin (HGB), mean corpuscular volume (MCV), mean corpuscular hemoglobin (MCH), mean corpuscular hemoglobin concentration (MCHC), thrombocytes (THR), heterophils (HE), eosinophils (EO), basophils (BA), lymphocytes (LY), and monocytes (MO).

Total white blood cell (WBC), red blood cell (RBC), and thrombocyte (THR) counts were obtained by manually counting cells using a hemocytometer following dilution with Natt-Herrick solution [37]. To achieve a 200 × dilution, 10 μL of whole blood from K_2_ EDTA tubes was mixed with 1,990 μL of Natt-Herrick solution. The mixture was allowed to stand for 20 minutes before 10 μL was loaded into each chamber of a Neubauer-improved Marienfeld hemocytometer (Paul Marienfeld GmbH, Germany). After settling for 5 minutes, white blood cells, red blood cells, and thrombocytes were manually counted under light microscopy at 200 × magnification. To minimize errors, each sample was counted twice, and the average was used. If the two counts differed by more than 10%, the sample was reanalyzed.

Differential leukocyte counts (HE, EO, BA, LY, and MO) were determined on Wright-Giemsa–stained blood smears. Under 1,000 × magnification with oil immersion microscopy, 100 leukocytes were identified and categorized into heterophils, eosinophils, basophils, lymphocytes, and monocytes. The absolute number of each leukocyte type was calculated by multiplying the total WBC count by the percentage obtained from the 100-cell differential count.

Hemoglobin concentration (HGB) was measured using the cyanmethemoglobin method. A mixture of 8 μL of K_2_ EDTA-treated whole blood and 2 mL of Drabkin’s reagent (ASAN SET Reagents for the Determination of Hemoglobin in Blood, Asanpharm, South Korea) was allowed to stand for 5 minutes, followed by centrifugation at 2,000 g for 5 minutes. The supernatant was placed into disposable cuvettes, and absorbance was measured at 540 nm using a spectrophotometer. Hemoglobin concentration was calculated by multiplying the absorbance by the concentration of a standard solution containing 16 mg/dL hemoglobin.

Packed cell volume (PCV) was determined by centrifuging K_2_ EDTA-treated whole blood in microhematocrit capillary tubes at 12,000 g for 5 minutes. PCV values were measured using a hematocrit reader card. MCV, MCH, and MCHC were calculated using standard formulas based on the manually determined values of RBC, HGB, and PCV.

**2) CBC using automated hematology analyzer**: CBC was also performed using the automated hematology analyzer (XN-1000V, Sysmex, Japan) with blood from K_2_ EDTA tubes, following to the manufacturer’s instructions. The XN-1000V incorporates both impedance and fluorescence flow cytometry technologies. Blood samples were analyzed using the “Bird” mode with the PLT-F channel, which applies fluorescence flow cytometry to distinguish nucleated blood cell populations in non-mammalian species. After analysis, users can utilize the Manual Analysis function to adjust cell classification. Since the default classification settings in the bird profile did not align with the scattergram patterns observed in duck blood, the classification gates in the PLT-F channel were modified based on cluster distributions identified in the first eleven samples. The scattergram was divided into three regions corresponding to RBC (including hemolyzed RBC), WBC, and platelets (PLT). In this study, PLT refers to the platelet count output by the analyzer, which in avian species corresponds to thrombocytes (THR). Thus, PLT values generated by the analyzer were compared with manually determined thrombocyte counts. HGB was measured using the SLS hemoglobin method, while HCT, MCV, MCH, MCHC, and RDW were calculated by the RBC channel using the adjusted profile. However, RDW was excluded from the comparative analysis result because it was not measured by the manual method.

**3) Biochemistry and electrolyte analysis:** Serum biochemistry and electrolyte tests were conducted using the automatic chemistry analyzer (Exdia PT10V, Precision Biosensor INC, South Korea) with the Comprehensive Plus 17V panel and Electrolyte 4V panels. The Comprehensive Plus 17V panel included albumin (ALB), alkaline phosphatase (ALP), alanine aminotransferase (ALT), amylase (AMY), blood urea nitrogen (BUN), calcium (Ca), cholesterol (CHOL), creatinine (CREA), gamma-glutamyl transferase (GGT), glucose (GLU), lipase (LIPA), phosphorus (PHOS), total bilirubin (TBIL), total protein (TP), albumin-to-globulin ratio (A/G ratio), blood urea nitrogen-to-creatinine ratio (B/C ratio), and globulin (GLOB). The Electrolyte 4V panel included sodium (Na), potassium (K), chloride (Cl), and the sodium-to-potassium ratio (Na/K ratio). These test panels were originally developed for use in mammals, and their validation in avian species has not yet been established. Since avian species were not included in the default species settings of the analyzer, the “Others” category was selected for analysis. All equipment operation and sample preparation were performed according to the manufacturer’s guidelines.

### 3. Genetic sex determination

Eastern spot-billed ducks lack external sexual dimorphism, requiring genetic analysis for sex determination. DNA was extracted from whole blood treated with K_2_ EDTA as an anticoagulant using the DNeasy Blood & Tissue Kit (Qiagen, Germany) according to the manufacturer’s instructions. PCR was performed to amplify the CHD-W gene located on the avian sex chromosome, using primers P2 (5′-TCTGCATCGCTAAATCCTTT-3′), P8 (5′-CTCCCAAGGATGAGRAAYTG-3′), and P0 (5′-ATTGAGTTGGAACCAGAICA-3′) [38]. The results of gene amplification were confirmed through electrophoresis.

### 4. Statistical analysis

#### 1) Calculation of reference intervals.

Reference intervals for hematology were calculated from values obtained by the manual method, whereas biochemical reference intervals were calculated from results of automated biochemical analyses, using the Reference Value Advisor software version 2.1 [39]. The statistical analysis in this study was performed in compliance with the guidelines established by the American Society for Veterinary Clinical Pathology (ASVCP) for determining reference intervals [21]. To identify potential outliers in the data, Tukey’s test and the Dixon-Reed test were applied. The distribution of the data was evaluated using histograms, Q-Q (quantile-quantile) plots, and the Anderson-Darling test. Based on the observed data distribution and sample size, different statistical methods were selected for calculating the reference intervals. Reference intervals were primarily calculated using the parametric method after applying Box-Cox transformation to approximate a Gaussian distribution. However, when the original data were already normally distributed and the transformation introduced distortion, the parametric method was instead applied to the untransformed data. For datasets that remained non-Gaussian even after Box-Cox transformation, the non-parametric bootstrap method was used.

#### 2) Comparison of manual and automated methods.

To compare the manual CBC results with those obtained from the XN-V automated hematology analyzer, statistical analyses, including paired t-tests, Pearson correlation analysis, and Bland-Altman plot analysis, were conducted using IBM SPSS Statistics software (version 28.0.0, IBM, USA). Paired t-tests were conducted to compare the mean values of the parameters between the two methods. Pearson correlation coefficients were calculated to assess the strength and direction of the relationship between measurements obtained using the manual and automated methods. Additionally, Bland-Altman plots were generated to evaluate the agreement between the two methods. A significance level of p < 0.05 was applied to determine whether the observed differences or correlations were statistically significant.

## Results

### 1. Reference intervals for hematologic and biochemical parameters.

**Fig 1:**
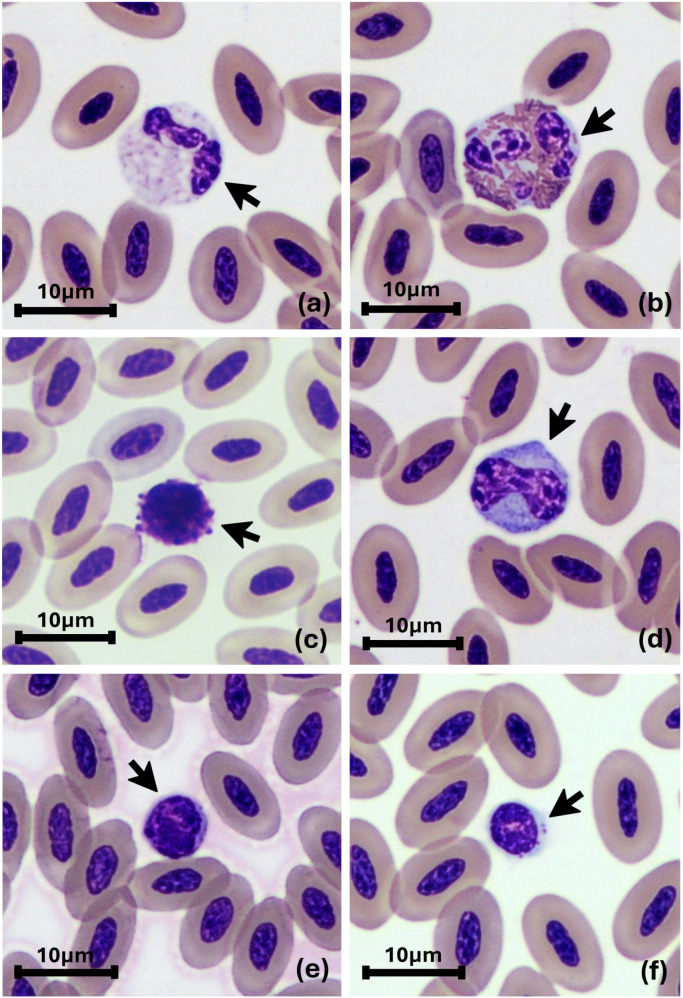
Blood cells of the Eastern spot-billed duck. Black arrows indicate the identified cells: (a) heterophil, (b) eosinophil, (c) basophil, (d) monocyte, (e) lymphocyte, (f) thrombocyte. Wright-Giemsa stained. 1000× with oil immersion.

Reference intervals were established for 13 hematologic and 18 biochemical parameters using data obtained from 55 clinically healthy juvenile Eastern spot-billed ducks (Tables 1 and 2). PLT and chemistry parameters were not measured in the first 11 samples, and three additional samples had missing values. Variation in sample size across parameters reflects the exclusion of statistical outliers, as detailed in the supplementary raw data (S1 Table). For each parameter, the final sample size, data distribution (Gaussian or non-Gaussian), and statistical method used (parametric, parametric after Box-Cox transformation, or non-parametric) are indicated in the corresponding results tables. The parameters BUN, BUN/Crea ratio, and GGT were excluded from the analysis due to the lack of measurable values and their limited clinical relevance in avian species. Sex determination identified 26 males and 29 females. No statistically significant differences were observed between males and females for any parameter; therefore, reference intervals were calculated using the combined dataset. The morphological characteristics of various blood cells were identified during the WBC differential process in Eastern spot-billed ducks (Fig 1).

**Table 1:** Reference intervals for hematology values of Eastern spot-billed duck based on manual analysis.

Parameters	n	Mean	SD	Min	Max	Reference Interval	90% CI for Lower Limit	90% CI for Upper Limit	Dist	Meth
WBC (10^3^/μL)	52	5.6	2.3	2.2	10.7	2.1–11.5	1.8 - 2.5	10.1 - 13.2	NG	TP
HE (%)	53	54.0	16.6	14.0	86.0	18.3–85.8	9.1 - 26.2	79.9 - 91.7	G	TP
EO (%)	53	13.7	8.2	3.0	36.0	2.5–33.5	0.0 - 3.3	28.5 - 38.9	NG	TP
BA (%)	53	0.2	0.4	0.0	2.0	0.0–1.7	0.0 - 0.0	1.0 - 2.0	NG	NP
LY (%)	53	18.2	13.4	2.0	54.0	2.8–63.0	1.9 - 3.8	46.9 - 83.0	NG	TP
MO (%)	53	13.8	5.5	4.0	26.0	3.3–25.6	1.1 - 5.2	23.0 - 28.1	G	TP
HE (10^3^/μL)	53	3.3	1.9	0.8	7.7	0.7–8.4	0.6 - 1.0	6.9 - 10.0	NG	P
EO (10^3^/μL)	53	0.8	0.6	0.1	4.0	0.1–2.6	0.1 - 0.2	1.9 - 3.5	NG	P
BA (10^3^/μL)	53	0.0	0.0	0.0	0.2	0.0–0.2	0.0 - 0.0	0.1 - 0.2	NG	NP
LY (10^3^/μL)	53	1.1	1.2	0.2	5.7	0.1–5.1	0.1 - 0.2	3.4 - 7.1	NG	P
MO (10^3^/μL)	53	0.8	0.4	0.2	2.0	0.2–1.9	0.2 - 1.9	1.6 - 2.2	NG	P
RBC (10^6^/μL)	54	3.1	0.4	2.3	4.1	2.4–3.8	2.2 - 2.5	3.7 - 4.0	G	P
HGB (g/dL)	49	12.7	1.6	9.2	15.6	9.1–15.7	8.1 - 10.0	15.1 - 16.2	G	TP
PCV (%)	51	45.6	3.3	38.0	52.0	38.9–52.3	37.7 - 40.1	51.0 - 53.6	G	P
MCV (fL)	53	148.5	17.0	111.6	187.3	116.9–185.6	111.9 - 122.5	177.7 - 194.5	G	TP
MCH (pg)	51	40.6	5.9	28.2	54.8	28.7–52.5	26.6 - 31.0	50.1 - 54.7	G	P
MCHC (g/dL)	50	27.6	3.2	20.2	33.9	19.9–33.1	17.5 - 22.0	32.2 - 34.0	G	TP
THR (10^3^/μL)	42	42.3	17.0	10.0	90.0	13.2–81.9	9.3 - 17.9	71.8 - 92.8	G	TP

SD, standard deviation; Dist, data distribution; G, Gaussian; NG, non-Gaussian; Meth, method; P, parametric; TP, parametric after box-cox transformation; NP, non-parametric; WBC, white blood cell; HE, heterophil; EO, eosinophil; BA, basophil; LY, lymphocyte; MO, monocyte; RBC, red blood cell; HGB, hemoglobin; PCV, packed cell volume; MCV, mean cell volume; MCH, mean corpuscular hemoglobin; MCHC, mean corpuscular hemoglobin concentration; THR, thrombocyte.

**Table 2:** Reference intervals for clinical biochemistry values of Eastern spot-billed duck using the Exdia PT10V analyzer.

Parameters	n	Mean	SD	Min	Max	Reference Interval	90% CI for Lower Limit	90% CI for Upper Limit	Dist	Meth
GLU (mg/dL)	43	254.7	29.6	185.0	310.0	197.1–317.6	186.6 - 209.5	300.9 - 332.1	G	TP
CREA (mg/dL)	43	0.2	0.1	0.1	0.4	0.1–0.4	0.1 - 0.1	0.3 - 0.4	NG	NP
Ca (mg/dL)	43	10.9	0.5	9.7	12.0	9.9–12.0	9.7 - 10.1	11.7 - 12.2	G	TP
PHOS (mg/dL)	43	5.0	1.2	2.7	7.7	2.6–7.3	2.0 - 3.1	6.8 - 7.8	G	P
TP (g/dL)	42	4.3	0.5	3.2	5.2	3.1–5.1	2.7 - 3.4	5.0 - 5.3	G	TP
ALB (g/dL)	43	1.4	0.2	1.0	1.8	1.0–1.8	1.0 - 1.0	1.6 - 1.8	NG	NP
GLOB (g/dL)	42	3.0	0.4	2.1	3.6	2.2–3.7	2.0 - 2.3	3.6 - 3.9	G	P
A/G ratio	43	0.5	0.1	0.3	0.6	0.3–0.6	0.3 - 0.4	0.6 - 0.6	NG	NP
ALT (U/L)	41	51.5	13.0	25.0	81.0	24.9–78.0	19.6 - 30.5	72.2 - 83.7	G	P
ALP (U/L)	42	456.2	141.5	219.0	882.0	253.7–844.2	232.0 - 281.3	708.6 - 1001.1	NG	TP
TBIL (mg/dL)	40	0.4	0.2	0.1	0.9	0.1–0.9	0.1 - 0.2	0.5 - 0.9	NG	NP
CHOL (mg/dL)	43	267.5	43.1	156.0	363.0	179.5–355.5	161.3 - 198.8	335.4 - 372.7	G	P
LIPA (U/L)	41	54.9	17.5	25.0	95.0	24.8–95.5	20.4 - 29.9	84.3 - 107.3	G	TP
AMY (U/L)	20	1860.2	431.4	1017.0	2432.0	934.9–2785.5	664.9 - 1232.7	2498.2 - 3104.9	G	P
Na (mmol/L)	42	143.2	4.5	132.0	150.0	132.1–149.9	132.0 - 136.3	148.0 - 150.0	NG	NP
K (mmol/L)	43	3.4	0.5	2.2	4.5	2.4–4.3	2.2 - 2.6	4.1 - 4.5	G	P
Na/K ratio	42	43.2	5.8	33.0	58.0	32.5–56.1	30.5 - 34.5	53.0 - 59.5	G	TP
Cl (mmol/L)	43	113.1	6.3	102.0	123.0	102.0–123.0	102.0 - 103.0	122.0 - 123.0	NG	NP

SD, standard deviation; Dist, data distribution; G, Gaussian; NG, non-Gaussian; Meth, calculating method; P, parametric; TP, parametric after box-cox transformation; NP, non-parametric; GLU, glucose; CREA, creatinine; Ca, calcium; PHOS, phosphorus; TP, total protein; ALB, albumin; GLOB, globulin; A/G ratio, albumin-to-globulin ratio; ALT, alanine aminotransferase; ALP, alkaline phosphatase; TBIL, total bilirubin; CHOL, cholesterol; LIPA, lipase; AMY, amylase; Na, sodium; K, potassium; Na/K ratio, sodium-to-potassium ratio; Cl, chloride.

### 2. Comparison of manual and automated hematology methods.

**Fig 2:**
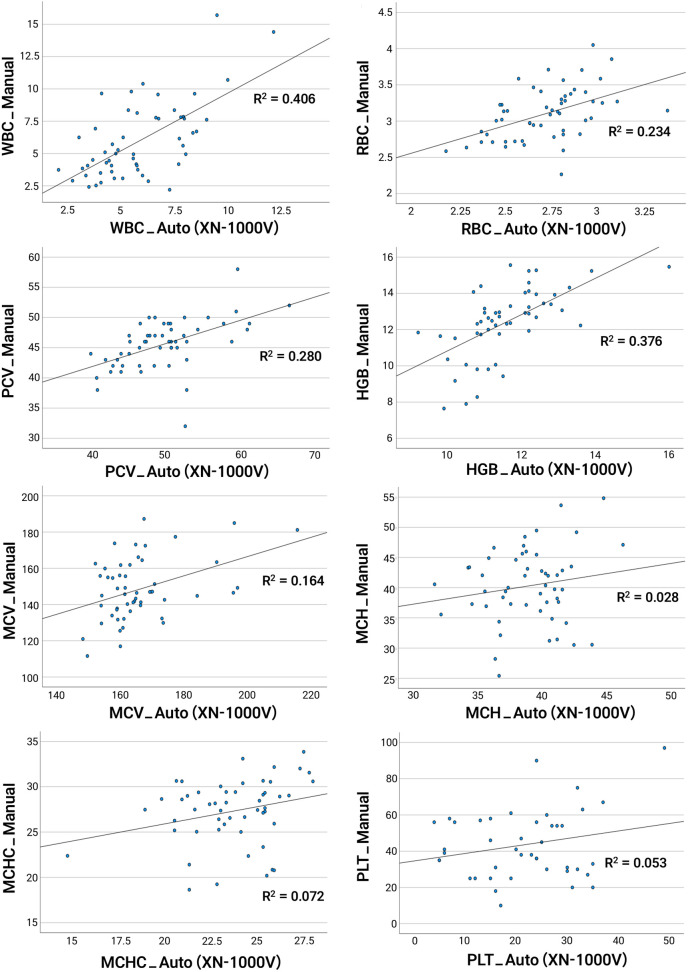
Scatter plots comparing manual and automated CBC parameters in Eastern spot-billed ducks. The x-axes represent the results from the automated CBC method, while the y-axes represent the results from the manual CBC method. The diagonal line in each plot indicates the regression line.

**Fig 3:**
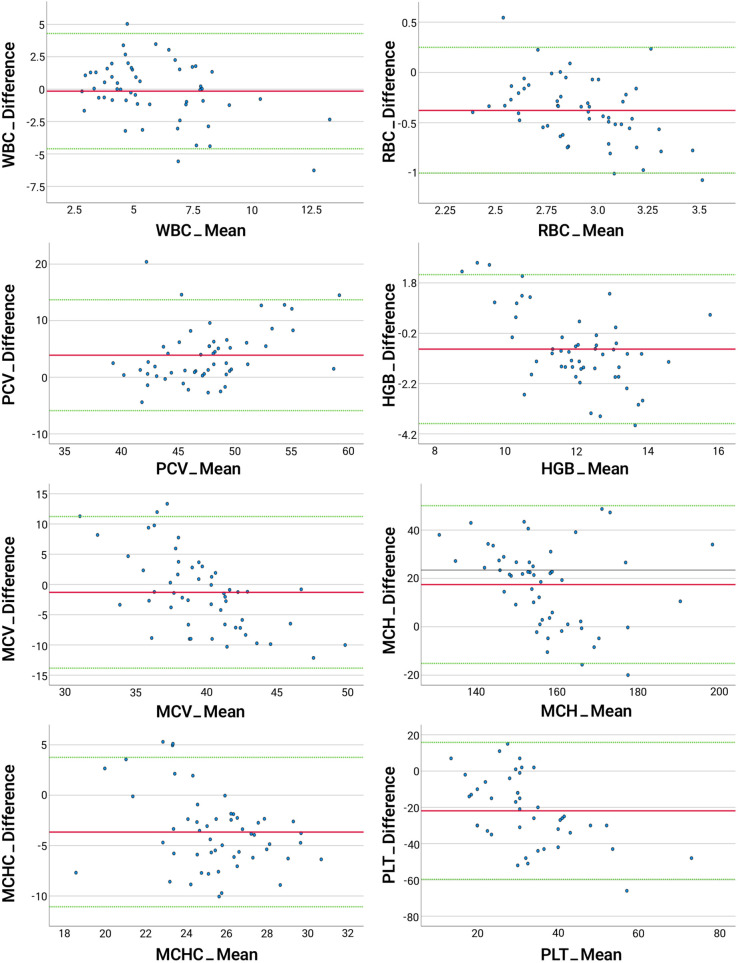
Bland-Altman plots comparing manual and automated CBC methods in Eastern spot-billed ducks. The x-axes represent the mean values obtained from both manual and automated methods, and the y-axes show the differences between the two methods for each hematological parameter. The red line represents the mean difference, with the green lines indicating the limits of agreement (±1.96 standard deviations).

Paired t-tests revealed statistically significant differences (p < 0.05) in mean values between manual and automated methods for most parameters, except for WBC and MCH (Table 3). Pearson correlation analysis demonstrated moderate correlations (p < 0.05, 0.4 < r < 0.7) for the majority of parameters, including WBC, RBC, HGB, PCV (HCT), and MCV, while no significant correlations (p > 0.05) were observed for MCH, MCHC, and THR (PLT) (Table 4, Fig 2). Bland-Altman analysis showed agreement between the two methods for most parameters, with a small number of outliers observed (Fig 3).

**Table 3:** Comparison of hematology parameters between manual and automated CBC methods in Eastern spot-billed ducks using paired t-test.

Parameters	n	Manual CBC		Automated CBC		Mean Difference	P value
		Mean	SD			Mean	SD
WBC (10^3^/μL)	54	5.96	2.93	5.81	2.07	0.2	0.629
RBC (10^6^/μL)	54	3.10	0.36	2.72	0.23	0.4	< 0.001
HGB (g/dL)	52	12.42	1.91	11.64	1.18	0.8	< 0.001
PCV (%)	53	45.58	4.14	49.67	5.84	3.9	< 0.001
MCV (fL)	53	148.44	16.98	165.87	12.76	17.5	< 0.001
MCH (pg)	52	40.29	6.16	38.94	3.02	1.3	0.150
MCHC (g/dL)	52	27.30	3.57	23.61	2.51	3.7	< 0.001
THR (10^3^/μL)	43	43.53	18.78	23.19	9.93	21.9	< 0.001

SD, standard deviation; WBC, white blood cell; HE, heterophil; EO, eosinophil; BA, basophil; LY, lymphocyte; MO, monocyte; RBC, red blood cell; HGB, hemoglobin; PCV, packed cell volume; MCV, mean cell volume; MCH, mean corpuscular hemoglobin; MCHC, mean corpuscular hemoglobin concentration; THR, thrombocyte. Automated CBC values were measured using the XN-1000V analyzer (Sysmex, Japan). HCT values obtained from the analyzer were compared with PCV, and PLT values were compared with THR.

**Table 4:** Pearson correlation analysis between manual and automated CBC methods in Eastern spot-billed ducks.

Parameters	Pearson Correlation (R)	P value
**WBC**	0.637	< 0.001
**RBC**	0.484	< 0.001
**HGB**	0.613	< 0.001
**PCV**	0.529	< 0.001
**MCV**	0.406	0.003
**MCH**	0.166	0.240
**MCHC**	0.269	0.054
**THR**	0.229	0.139

SD, standard deviation; WBC, white blood cell; HE, heterophil; EO, eosinophil; BA, basophil; LY, lymphocyte; MO, monocyte; RBC, red blood cell; HGB, hemoglobin; PCV, packed cell volume; MCV, mean cell volume; MCH, mean corpuscular hemoglobin; MCHC, mean corpuscular hemoglobin concentration; THR, thrombocyte. Automated CBC values were measured using the XN-1000V analyzer (Sysmex, Japan).

## Discussion

The established hematologic and biochemical reference intervals for juvenile Eastern spot-billed ducks were compared with previously reported values for other *Anas* species, including mallard and domestic duck, black duck (*Anas superciliosa*), and mottled duck (*Anas fulvigula*) (Tables 5 and 6) [11,15–20]. None of the cited studies fully adhered to ASVCP guidelines, and many presented only means and standard deviations due to limited sample sizes. Therefore, comparisons were made using these summary statistics. While the overall values were generally comparable, direct comparisons should be interpreted with caution, as differences in study design—including the use of wild-caught versus captive individuals, unreported husbandry conditions, and the inclusion of adults only—introduce considerable variability across datasets.

**Table 5:** Comparison of mean and standard deviation of hematological parameters in Eastern spot-billed ducks and related species.

Parameters	Eastern spot-billed duck (*A. zonorhyncha*) (n = 54)	Khaki Campbell duck (*A. platyrhynchos domesticus)*^a^ (n = 10)	Indian runner duck (*A. platyrhynchos domesticus)*^b^ (n = 10)	Mottled duck (*A. fulvigula*)^c^ (n = 81)	Black duck (*A. superciliosa)*^d^ (n = 17)
WBC (10^3^/μL)	5.6 ± 2.3	12.2 ± 1.4	6.3 ± 0.3	20.9 ± 0.8	19.7 ± 6.6
HE (%)	54.0 ± 16.6	16.3 ± 3.9	23.0 ± 3.7	–	–
EO (%)	13.7 ± 8.2	36.9 ± 7.4	10.8 ± 0.6	–	–
BA (%)	0.2 ± 0.4	2.6 ± 0.7	2.0 ± 0.5	–	–
LY (%)	18.2 ± 13.4	36.8 ± 5.5	41.3 ± 3.1	–	–
MO (%)	13.8 ± 5.5	3.6 ± 1.9	22.9 ± 1.9	–	–
RBC (10^6^/μL)	3.1 ± 0.4	2.1 ± 0.1	2.5 ± 0.1	–	2.8 ± 0.2
HGB (g/dL)	12.7 ± 1.6	9.4 ± 0.8	13.8 ± 0.3	12.5 ± 2.0	13.0 ± 1.4
PCV (%)	45.6 ± 3.3	34.4 ± 1.48	41.2 ± 1.4	45.0 ± 0.6	40.2 ± 4.3
MCV (fL)	148.5 ± 17.0	167.3 ± 9.7	192.2 ± 7.7	–	144.7 ± 10.0
MCH (pg)	40.6 ± 5.9	44.5 ± 2.0	64.6 ± 2.1	–	46.6 ± 3.0
MCHC (g/dL)	27.6 ± 3.2	27.6 ± 2.3	33.6 ± 1.1	–	32.2 ± 1.2

WBC, white blood cell; HE, heterophil; EO, eosinophil; BA, basophil; LY, lymphocyte; MO, monocyte; RBC, red blood cell; HGB, hemoglobin; PCV, packed cell volume; MCV, mean cell volume; MCH, mean corpuscular hemoglobin; MCHC, mean corpuscular hemoglobin concentration; THR, thrombocyte; a, Data from Bhattacherjee et al., 2018 (growers, male); b, Data from Dalai et al., 2015 (adult, female); c, Data from Ratliff et al., 2017 (adult, all sexes included); d, Data from Mulley, 1979 (all ages and sexes included) [11,16,18,19].

**Table 6:** Comparison of mean and standard deviation of biochemical parameters in Eastern spot-billed ducks and related species.

Parameters	Eastern spot billed duck *(A. zonorhyncha)* (n = 43)	Mallard *(A. platyrhynchos)*^a^ (n = 13)	Indian runner duck *(A. p. domesticus)*^b^ (n = 8)	Mottled duck (*A. fulvigula*)^c^ (n = 82)	Black duck (*A. superciliosa)*^d^ (n = 15)
GLU (mg/dL)	254.7 ± 29.6	185.0 ± 47.0	220.5 ± 19.0	253.0 ± 5.6	175.8 ± 26.5
CREA (mg/dL)	0.2 ± 0.1	0.3 ± 0.1	–	–	–
Ca (mg/dL)	10.9 ± 0.5	9.4 ± 1.9	14.8 ± 5.6	–	–
PHOS (mg/dL)	5.0 ± 1.2	2.9 ± 1.0	–	–	3.2 ± 1.2
TP (g/dL)	4.3 ± 0.5	3.8 ± 0.7	4.3 ± 0.9	–	4.3 ± 0.4
ALB (g/dL)	1.4 ± 0.2	1.5 ± 0.4	1.7 ± 0.4	–	3.0 ± 0.3
GLOB (g/dL)	3.0 ± 0.4	–	2.6 ± 0.5	–	–
A/G ratio	0.5 ± 0.1	–	0.6 ± 0.1	–	2.7 ± 0.8
ALT (U/L)	51.5 ± 13.0	26.3 ± 8.0	24.8 ± 5.2	–	–
ALP (U/L)	456.2 ± 141.5	26.3 ± 8.0	117.0 ± 51.9	–	20.9 ± 11.7
TBIL (mg/dL)	0.4 ± 0.2	0.16 ± 0.05	–	–	0.3 ± 0.0
Na (mmol/L)	143.2 ± 4.5	–	141.3 ± 2.1	146.0 ± 0.3	–
K (mmol/L)	3.4 ± 0.5	–	2.8 ± 0.4	3.9 ± 0.1	–
Cl (mmol/L)	113.1 ± 6.3	–	105.3 ± 1.7	111.0 ± 0.3	–

GLU, glucose; CREA, creatinine; Ca, calcium; PHOS, phosphorus; TP, total protein; ALB, albumin; GLOB, globulin; A/G ratio, albumin-to-globulin ratio; ALT, alanine aminotransferase; ALP, alkaline phosphatase; TBIL, total bilirubin; CHOL, cholesterol; LIPA, lipase; AMY, amylase; Na, sodium; K, potassium; Na/K ratio, sodium-to-potassium ratio; Cl, chloride; a, Data from Fairbrother et al., 1990 (adult, male); b, Data from Franco et al., 2010 (unknown); c, Data from Ratliff et al., 2017 (adult, all sexes included); d, Data from Mulley, 1979 (all ages and sexes included) [15,18–20].

While most parameters showed no significant differences compared to other *Anas* species, heterophils were predominant in the WBC differential of Eastern spot-billed ducks, in contrast to the lymphocyte dominance typically observed in other *Anas* species [16–18]. This heterophil-dominant leukogram has also been observed in mottled ducks and ferruginous ducks (*Aythya nyroca*) [19,40]. This variation in the leukogram may reflect a species-specific characteristic of Eastern spot-billed ducks. However, considerable individual variability was observed in leukograms, and the potential influence of stress related to capture and handling cannot be entirely excluded [41]. Although the ducks in this study were housed in outdoor enclosures and received regular feeding, they originated from wild populations and were reared for release, with minimal human contact apart from feeding and weekly weighing. As such, they may have been more reactive to capture than typical captive birds. In great tits (*Parus major*), leukogram changes including increased heterophils and decreased lymphocytes and eosinophils have been observed within 30–120 minutes post-capture [42]. In our study, some individuals exhibited agitation during net capture, which may have resulted from being chased within large outdoor enclosures. Although an acclimation period was provided to allow the ducks to settle before sampling, the duration was neither standardized nor recorded. Leukogram changes associated with stress may have become more pronounced during this period. Therefore, additional studies with standardized handling protocols are needed to better assess the impact of acute stress on hematologic values in this species.

The morphology of blood cells in Eastern spot-billed ducks was examined, revealing similarities to other Anseriformes [25]. Eosinophil granules in Eastern spot-billed ducks exhibited a distinctly pronounced rod shape, more prominent than the typical round-to-oval shaped granules in mallards and Pekin ducks [25,43]. The origin of the distinctive granule morphology observed in ducks has not yet been elucidated through ultrastructural studies [25].

In the biochemistry results, several notable findings were observed. Amylase levels exceeded the analyzer’s maximum measurable range of 2500 U/L in several samples, resulting in the exclusion of these data from the reference interval calculation. Amylase elevations are typically associated with acute pancreatitis and enteritis; however, previous researches on mallards and Pekin ducks suggest that normal amylase levels in *Anas* species may naturally exceed this threshold [20,44]. The Exdia PT10V analyzer utilized in this study employs the saccharogenic method for measuring amylase. In dogs, this method is susceptible to interference from glucoamylase and maltase, potentially resulting in falsely elevated values, and the amyloclastic method is preferred for greater accuracy [45]. Whether similar interference occurs in birds remains unknown, requiring further investigation to accurately measure amylase through additional testing using diluted samples, alternative analyzers, or different measurement methods. Elevated alkaline phosphatase (ALP) levels were also observed, likely due to the young age of the subjects, as bone growth was not yet complete [46]. Additionally, mammalian biochemical test kits were used, which may not fully reflect the diagnostic needs of avian species. Consequently, parameters such as aspartate aminotransferase (AST), uric acid, and bile acids, important for clinical assessment in birds, were not measured [46]. This limitation highlights the need for further studies to establish reference intervals for these parameters in this species.

Statistical analysis comparing the manual CBC method and the automated hematology analyzer revealed both agreements and discrepancies across parameters. Thrombocyte counts showed notable discrepancies and were consistently underestimated by the automated method. In contrast, the differences observed for parameters such as WBC, RBC, HGB, PCV, and MCV, although statistically significant, were generally within ranges not considered clinically relevant. Moderate correlations were also observed for these parameters, and Bland-Altman analysis indicated that most values fell within acceptable limits of agreement with relatively few outliers. These findings suggest that the automated analyzer may have clinical utility for several hematological parameters in Eastern spot-billed ducks, but its limitations, particularly for thrombocyte counts, should be carefully considered. for most parameters, supporting the clinical applicability of the automated analyzer.

The weak correlations observed for MCH, MCHC, and thrombocyte, along with significant mean differences in thrombocyte counts, may stem from inherent variability in hematology testing. In clinical laboratory medicine, allowable total errors (TEa), refers to the maximum deviation from the true value that is considered clinically acceptable [47]. According to the ASVCP guidelines, TEa thresholds for RBC, hemoglobin, and MCHC are approximately 10%, while WBC counts may be allowed up to 15–20%, and platelet counts up to 20–25%, depending on laboratory conditions [47]. This variability may be further exacerbated in cases of rapid aggregation or inherently low thrombocyte counts [47]. While imprecision and bias were not directly assessed through repeat measurements in this study, and these TEa thresholds were originally derived from mammalian data, the observed variability in thrombocyte measurements may nonetheless reflect the intrinsic limitations of accurately quantifying these cells. Additional discrepancies may result from the rapid degradation of avian whole blood, even under refrigerated conditions, as well as potential issues such as incomplete anticoagulation or hemolysis caused by anticoagulant [26,48–50]. In this study, all blood tests were conducted within 12 hours of collection to mitigate potential issues, however, variations in the time between collection and analysis may still have contributed to sample degradation. As storage time was not consistently recorded, we were unable to evaluate its potential effects on the results. Further studies are needed to clarify the impact of time between sampling and analysis on hematologic and biochemical parameters.

Additionally, the automated analyzer requires user-defined scattergram settings for each species, which involve manually dividing cell distribution regions in the PLT-F channel scattergram. However, the lack of clear boundaries between cell populations can lead to significant variability in results depending on the extent of RBC inclusion in the defined regions, and errors in these settings may have contributed to the observed discrepancies. Similar challenges related to scattergram-based cell classification have been noted in other studies using the XN-1000V for non-mammalian species. Meazzi et al. analyzed blood samples from Hermann’s tortoises using the WNR channel, which is designed for nucleated RBCs, under the “Dolphin” species setting [35]. They classified cells into only two broad categories—RBC and a combined group of WBC and thrombocytes—based on the observed scattergram clusters. In contrast, Mesalles et al. conducted hematologic analysis in rainbow trout using the PLT-F channel under the “Bird” mode, as in the present study [34]. Given the difficulty in distinguishing thrombocytes from lymphocytes, they reclassified the non-RBC population into two groups: heterophils and mononuclear cells. Notably, greater inconsistency was reported in the heterophil group, possibly due to the small number of cells and the difficulty in clearly distinguishing clusters within the scattergram.

Moreover, the manual method was used as a comparison group to validate the automated analyzer. During blood cell counting, the difficulty in distinguishing lymphocytes and thrombocytes, which share similar shapes and sizes, may have introduced human error, potentially impacting the results. If further research is conducted, this limitation could be addressed by incorporating cross-validation techniques using staining agents such as Phloxine B dye to improve the accuracy of the analysis [36].

In this study, no sex-related differences were observed in either hematology or biochemical parameters, consistent with findings from other studies on *Anas* species [17–19]. According to a recent study on forest bird species, the H/L ratio can be influenced by both sex and age, with higher values generally observed in adults compared to juveniles, and in females compared to males [24]. As the study’s sample population was limited to juvenile individuals, future studies are needed to include data from sexually mature adult ducks to better evaluate the influence of age and sex on these parameters. Furthermore, since the ducks in this study were reared in captivity for 3–4 months prior to sampling, potential hematologic differences from wild counterparts should be taken into account when interpreting the results.

## Conclusions

Species-specific hematologic and biochemical reference intervals for Eastern spot-billed ducks (*Anas zonorhyncha*) were established for the first time through this study, providing valuable baseline data for monitoring health and diagnosing diseases in this species. However, as the study was limited to juveniles reared under semi-captive conditions, caution is advised when applying these reference values to wild or adult populations. Moreover, this study validated the avian blood analysis capabilities of the XN-1000V automated hematology analyzer for the first time. Although the results were not completely consistent with those obtained using the manual method, they exhibited minor discrepancies unlikely to affect clinical diagnosis and demonstrated moderate correlations in most parameters between the two methods, supporting its suitability for clinical diagnostics. Especially, compared to the traditional manual method, which is time-consuming and prone to errors, the automated method offers significant advantages in terms of speed, simplicity, and accessibility, making it highly practical for clinical applications. Continued refinement of analyzer settings and validation across diverse avian taxa will be essential to improve diagnostic accuracy and expand clinical relevance.

## Supporting information

S1 Table Raw hematology and biochemistry data from 55 juvenile Eastern spot-billed ducks. Values excluded as outliers in reference interval analysis are highlighted in red.
(XLSX)
